# Additive Manufacturing of Bioactive Glass and Its Polymer Composites as Bone Tissue Engineering Scaffolds: A Review

**DOI:** 10.3390/bioengineering10060672

**Published:** 2023-06-01

**Authors:** Lizhe He, Jun Yin, Xiang Gao

**Affiliations:** 1 Center for Medical and Engineering Innovation, The First Affiliated Hospital of Ningbo University, Ningbo 315010, China; hlznew@live.com; 2 The State Key Laboratory of Fluid Power Transmission and Control Systems, Key Laboratory of 3D Printing Process and Equipment of Zhejiang Province, School of Mechanical Engineering, Zhejiang University, Hangzhou 310028, China; 3 Department of Neurosurgery, The First Affiliated Hospital of Ningbo University, Ningbo 315010, China

**Keywords:** additive manufacturing, bioactive glass, bone tissue engineering, scaffold

## Abstract

Bioactive glass (BG) and its polymer composites have demonstrated great potential as scaffolds for bone defect healing. Nonetheless, processing these materials into complex geometry to achieve either anatomy-fitting designs or the desired degradation behavior remains challenging. Additive manufacturing (AM) enables the fabrication of BG and BG/polymer objects with well-defined shapes and intricate porous structures. This work reviewed the recent advancements made in the AM of BG and BG/polymer composite scaffolds intended for bone tissue engineering. A literature search was performed using the Scopus database to include publications relevant to this topic. The properties of BG based on different inorganic glass formers, as well as BG/polymer composites, are first introduced. Melt extrusion, direct ink writing, powder bed fusion, and vat photopolymerization are AM technologies that are compatible with BG or BG/polymer processing and were reviewed in terms of their recent advances. The value of AM in the fabrication of BG or BG/polymer composites lies in its ability to produce scaffolds with patient-specific designs and the on-demand spatial distribution of biomaterials, both contributing to effective bone defect healing, as demonstrated by in vivo studies. Based on the relationships among structure, physiochemical properties, and biological function, AM-fabricated BG or BG/polymer composite scaffolds are valuable for achieving safer and more efficient bone defect healing in the future.

## 1. Introduction

The fracture, infection, and surgical removal of bones may all lead to the development of bone defects. With the number of new incidences of bone fracture having increased by 33.4% in the past three decades, the clinical and economic burden associated with bone defects has reasonably grown with time [1]. The incidence of bone defects results not only in chronic pain but, more importantly, in the loss of bone function (for example, the production of blood cells, the maintenance of mineral homeostasis, weight-bearing capacity, the assistance of limb movement, and the protection of organs/soft tissues by the cranium, ribs, and vertebrae) [2]. Nevertheless, the incidence of bone defects significantly impairs patients’ quality of life [3].

Once damaged, the bones can initiate self-mediated regeneration. Nonetheless, when the size of the bone defect is beyond a critical size, for example, 1–2 cm or 50% of the circumvent of the bones, spontaneous bone regeneration cannot occur [3]. Autologous bone transplantation remains the gold-standard therapy for critically sized bone defects, which involves transplanting the patient’s own bone to the defect site. Nonetheless, this technique involves numerous drawbacks, including limited donor bone availability, requiring additional surgeries to harvest the bone, and donor site morbidity [4]. Bone grafts sourced from other patients (allograft) or species other than humans (xenograft) are also used, but they are linked to the issues of infection, immunogenicity, foreign body reactions, chronic inflammation, and slower incorporation into local bones [5]. This situation calls for the research and development of synthetic materials that physically fill and support the bone defects, exhibiting excellent biocompatibility and, more importantly, an ability to support the adhesion, proliferation, and osteogenic functions of specific cell types (e.g., mesenchymal stem cells, (pre)osteoblasts). The ultimate goal when using these materials, also known as tissue engineering scaffolds, is to induce the formation of new bone that finally restores the defective bone’s structure and function.

With the demand clarified, numerous materials have been developed and tested for their potential in bone defect repair. Metals and polymers that are biologically inert, minimally resorbable and provoke low toxic responses/foreign body reactions have been investigated since the 1960s [6]. Nonetheless, these materials failed to bond intimately to the surrounding tissues. Calcium phosphate ceramics (i.e., hydroxyapatite, β-tricalcium phosphate, and their binary mixtures) are among the second generation of biomaterials investigated for bone defect repair [7]. These materials can bond with the surrounding bone tissues but are less capable of stimulating the regeneration of vascular networks. From 1969 to 1971, studies led by Prof. Larry Hench at the University of Florida reported that the partly or fully amorphous “45S5.0” glass–ceramic, which comprised 45% SiO_2_, 24.5% Na_2_O, 24.5% CaO, and 6% P_2_O_5_ (all in weight percentage), bonded intimately to a rat femur 6 weeks after the implantation [8,9]. When compared to calcium phosphate ceramics, 45S5.0 (later known as 45S5 Bioglass) binds to bones more efficiently, offering additional benefits of binding to the soft tissues, as well as inducing angiogenesis, which plays a vital role in the full regeneration of highly vascularized bone tissues [10,11]. The emergence of bioactive glass (BG) has ignited research interest globally, as evidenced by the continuous growth in the number of publications since 1971. Much effort has been made to unveil the mechanism of bioactivity [9,12,13], the relationships among compositional, structural, and physiochemical properties [14,15,16], novel glass synthesis methods [17,18,19], and the functionalization of BG [20,21,22], with the aim of achieving safer and more effective bone tissue regeneration induced by BG scaffolds.

Nonetheless, very little attention has been paid to processing BG into the desired geometry, which plays an important role in determining the performance of a scaffold. A contour matching the anatomy of defect sites allows for the press-fit implantation of biomaterials, thereby facilitating the implantation process [23]. More importantly, a well-defined interconnecting porosity is essential for the ingrowth of vascularized bone tissues, which, meanwhile, determines the mechanical and degradational properties of the BG. However, the traditional machining of BG is rather challenging owing to the low fracture toughness along with the high Young’s modulus of BG [24]. Hence, the relevant strategy is to prepare BG/polymer composites, where BG particles offer the composites biological functions and the polymer phase contributes to better malleability and processibility. However, the conventional techniques of producing porous polymer composites, that is, phase separation, porogen templating, and gas foaming, may not guarantee on-demand configuration of the size, shape, volumetric ratio, spatial distribution, and interconnectivity of pores.

Additive manufacturing (AM), more commonly known as “3D printing”, has been proposed as a solution to the aforementioned issue. Rather than being removed, materials are added onto the building object on-demand in a layer-by-layer manner, and the entire manufacturing process is automated and program-controlled [25]. The benefits of AM in the fabrication of BG or BG/polymer tissue engineering scaffolds can be seen in multiple aspects, which include, but are not limited to, yielding a high-precision, patient-specific geometry, and accurate control of the intricate porous structure [26]. Taken together, the application of AM potentially allows BG or BG/polymer tissue engineering scaffolds to satisfy the need for patient-specific anatomic fitting and well-defined biological function that is dependent on the structure–property relationship, thereby achieving safer and more efficient bone defect healing.

Meanwhile, the past two decades have witnessed the increasing application of AM in the fabrication of bone tissue engineering scaffolds. Early in 2001, Hutmacher reviewed multiple additive manufacturing technologies for their application in scaffold fabrication, emphasizing their potential of becoming the “*most important tools for tissue engineering in the future*”, so that biomaterials chosen for scaffold fabrication shall be compatible with these technologies [27]. The advances in the architecture design and application of specific AM technologies in the context of tissue engineering scaffolds are summarized elsewhere [28,29,30,31,32]. Regarding biomaterials, however, while the research progress of AM-fabricated metal [33], ceramic [34], and natural polymers [35], as well as of metal/polymer [36] and mineral/polymer composites [37], as bone tissue engineering scaffolds has been summarized elsewhere, literature reviews of additive-manufactured BG and BG/polymer composites are less available.

This work reviewed the advances made in the field of additive manufacturing with BG. A literature search was performed in Scopus database using the query: (TITLE-ABS-KEY(additive manufactur*) OR TITLE-ABS-KEY(3D print*) OR TITLE-ABS-KEY(rapid prototyp*)) AND (TITLE-ABS-KEY(Bioglass) OR TITLE-ABS-KEY(bioactive glass) OR TITLE-ABS-KEY(phosphate glass) OR TITLE-ABS-KEY(borate glass)). Journal publications and book chapters in English that were published before 6 April 2023 were included. Careful screening was performed by the author (L.H.) to exclude publications that were not relevant, including those that: (1) reported the formation of BG or BG/polymer structures upon an AM-fabricated mold/template that was not composed of BG or BG/polymer; (2) reported the crystallization of BG during AM or after post-AM processing, unless they were cited on purpose for a discussion related to the crystallization of BG in AM.

This work begins with an introduction to the material properties of the major types of BG and BG/polymer composites. Next, the work focuses on introducing various technologies that have been applied for the AM of these materials so far. The mechanism, the requirements of feedstock preparation, and the drawbacks of each technology have been summarized. Cases where the additive-manufactured BG and BG/polymer composites were applied in in vivo bone defect treatment are then discussed to demonstrate the value and potential of using AM to fabricate BG-containing scaffolds. Finally, we offer our perspectives on what future research should focus on.

## 2. BG and BG/Polymer Composites Typically Applied in Bone Tissue Engineering

Before reviewing how additive-manufactured BG and BG/polymer composites are utilized to treat bone defects, a brief introduction of typical BG and BG/polymer composites and their compositional and functional characteristics is necessary. Depending on the forming units of the glass network, BG used in bone tissue engineering is conventionally classified as silicate-, phosphate-, and borate-based BG. While bulk BG can be applied in bulk form for bone defect healing, a more pragmatic practice would be using BG/polymer composites as they offer superior ductility and plasticity.

### 2.1. Bioactive Glasses (BG)

#### 2.1.1. Silicate-Based BG

Silicate-based BG is the first type of BG ever developed, dating back to the late 1960s [8]. The primary structural unit of the glass network is a SiO_4_ tetrahedron, which links to each other via Si-O-Si covalent bonds to form the glass network. Meanwhile, monovalent or divalent cations of alkali/alkaline earth elements “modify” the glass network by ionically bonding to the oxygen atoms. This event leads to reduced bridging oxygens (Ø, referring to oxygens within Si-O-Si covalent bonds) in the glass network. As a result, the network connectivity, as determined by the average number of bridging oxygens in each SiO_4_ tetrahedron, reduces. This change leads to lowered chemical stability, increased solubility, as well as a reduced temperature of softening and melting [38].

Conventionally, silicate-based BG is synthesized through a melt-quenching route. The selected oxides in BG are homogenized, followed by heating at >1000 °C to process the oxides into a homogenous liquid glass melt, which is then cast into a mold or water to yield solid glass that is generally non-porous [39]. Since 1991, the sol-gel process has also been developed for the synthesis of silicate-based BGs [17]. The organic precursors (e.g., tetraethyl orthosilicate) are hydrolyzed in an acidic solution. The mixture is then homogenized with calcium-containing compounds (e.g., CaNO_3_·4H_2_O), and undergoes polycondensation to yield a silica network in gel form, with the water contained later evaporated. This dried gel is finally heat-treated at 500–700 °C to obtain a solid glass. A unique feature of the sol-gel process is that the resultant glass possesses a mesoporous microstructure that can be exploited for the delivery of drugs and bioactive molecules (e.g., cytokine [40], growth factor [41], and siRNA [42]). This mesoporous glass structure also yields a greater surface area, thereby improving the solubility and apatite formation ability of the BG relative to those of its melt-quenched counterparts [39,43]. With the addition of non-ionic surfactants (e.g., Pluronic P123, F127, or cetrimonium bromide) during the sol-gel process, a supramolecular arrangement of the silicate–organic complex is formed, which forms a highly ordered mesoporous BG structure. Such a mesoporous structure allows for more homogeneous loading and the release of molecules [18,44,45].

The mechanism of bone-bonding bioactivity involves a series of activities, as elaborated by Prof Larry Hench [9,46]:

1. Alkali metal cations within glass exchange with H^+^/H_3_O^+^ in the surrounding medium (Si-O-M + H^+^ → Si-OH + M^+^).
2. Hydrolytic attacks take place at the Si-O-Si bonds within the soluble SiO_2_, giving rise to hydrated silica (Si-OH) at the BG–liquid interface [38].
3. The hydrated silica undergoes polycondensation and repolymerization, which results in the formation of a silica-rich gel layer, as well as the depletion of metal cations from the BG.
4. Ca^2+^, PO_4_^3−^, and CO_3_^2−^ presented in the aqueous environment migrate to and become absorbed by the silica-rich layer, forming an amorphous layer of calcium phosphate [7,47].
5. The calcium phosphate layer crystallizes by incorporating OH^−^ and CO_3_^2−^ from the surrounding medium to form a hydroxycarbonate apatite (HCA) layer.

The HCA layer subsequently enables the adsorption of biological moieties, which modulate the attachment, proliferation, and osteogenic differentiation of stem cells. Eventually, the cells committed to osteogenesis produce a bone matrix and lead to new bone formation [47].

**Figure 1:**
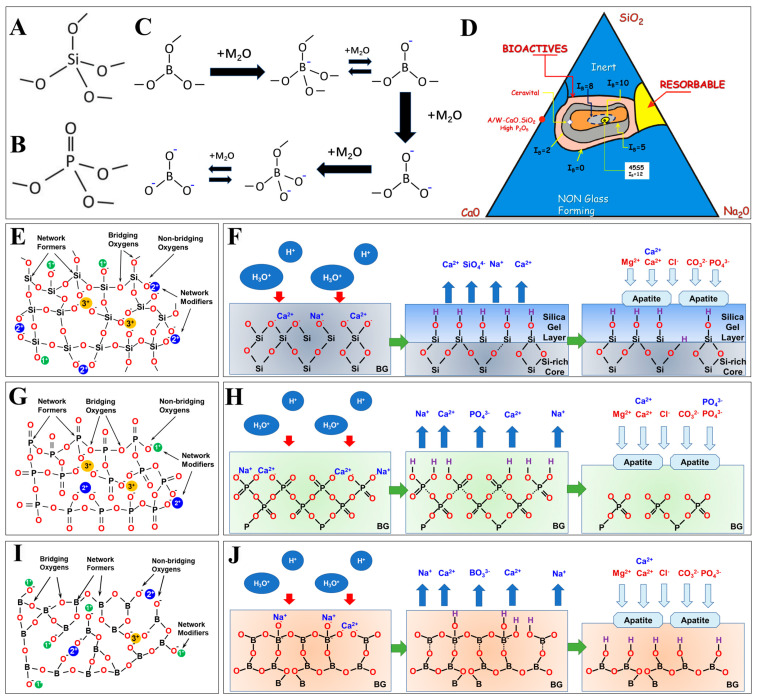
A summary of structural properties and mechanisms of degradation as well as bone-binding bioactivity for various BG species. (**A**) Tetrahedral SiO_4_. (**B**) Tetrahedral PO_4_. (**C**) The transition of borate units in a borate-based glass along with an increased metal oxide ratio. (**D**) Relationship between the composition of Si-P-Ca-Na glass–ceramic (P = 6 wt.%) and index of bioactivity (I_b_), defined as the inverse of the time required for bonding formation at >50% of the bone–BG interface. Reprinted from references [73]. (**E**–**J**) Schematic diagram of glass network and the mechanism of hydrolytic degradation as well as bone-binding bioactivity of (**E**,**F**) silicate-based BG, (**G**,**H**) phosphate-based BG, and (**I**,**J**) borate-based BG. Reprinted and adapted from references [74,75].

The five steps of HCA crystallization are regarded as the basis of the bioactivity of BG, and are dependent on the BG chemical formulation to a great extent. Typically, BG contains high CaO content and <60 mol.% of SiO_2_, as shown in Figure 1D [10]. Based on this diagram, more BG formulas (Table 1) are developed with variations in the type and ratio of metal oxides in the glass composition, with some formulas approved and commercialized for clinical application.

An evident drawback of silicate-based BG is the incomplete conversion of glass into bioactive apatite. As mentioned previously, a silica gel layer is formed at the surface of the BG in body fluid. Such a gel layer only allows metal cations from the glass to be leached into the surrounding medium, but protects the inner region of the BG from dissolution [48]. Consequently, the glass beneath the silica gel layer converts into a silica-rich phase that cannot be replaced by newly formed bone tissues. It has been reported that the residual silica with a submicron size induces cytotoxicity and chronic inflammation, and the accumulation of silica in the spleen and liver may induce further complications [49,50].

**Table 1:** Representative silicate-based BG.

Code	Composition (Oxides of Each Element)	Remark	Refs.
45S5	45Si–6P–24.5Na–24.5 Ca (wt.%)	Commercialized as NovaMin^®^ (GSK plc, London, UK)	[9]
13–93	53Si-4P-6Na-5Mg-12K-20Ca (wt.%)	-	[51]
S53P4	53Si–4P–23Na–20Ca (wt.%)	Commercialized as Bonalive^®^ (Bonalive Biomaterials Ltd., Turku, Finland)	[52]
SP-17Sr-17Ca	44.5Si–4.5P–4Na–7.5Mg–4K–17.8Ca–17.8Sr (mol.%)	-	[53,54]
58S	60Si–4–36Ca (mol.%)	-	[39]
Si70-Ca30	70Si–30Ca	Commercialized as TheraGlass^®^ (TheraGlass Ltd., London, UK)	[55]

#### 2.1.2. Phosphate-Based BG

Owing to a composition similar to the mineral phase of bone, phosphate-based BG with a high calcium content has received much attention as a biomaterial targeted toward bone-related applications. The glass network of a phosphate-based glass is primarily formed by interconnecting PO_4_ tetrahedrons, which contain a central phosphorous atom, a double-bonded oxygen atom, and three single-bonded oxygen atoms. The single-bonded oxygen atoms either serve as bridging oxygen (Ø) in the covalent P-O-P bonds, or non-bridging oxygens when ionically linked to the alkali/alkaline earth metal cation [38]. In contrast to silicate-based glass, where a high silica content contributes to the chemical stability of the glass, P_2_O_5_ is highly reactive and hygroscopic, thereby contributing to the good solubility of phosphate glass [38]. While the conversion of bridging oxygens into non-bridging oxygens occurs as metal oxides are added to the glass network, a reduced number of P-O-P bonds typically contributes to the increased chemical stability of the glass, which is more pronounced for cations with a greater valency and a higher charge-to-size ratio [56].

Unlike silicate-based BG, phosphate-based BG is fully degradable in an aqueous environment. After an ionic exchange of metal cations and H^+^/H_3_O^+^, the P-O bonds are gradually hydrolyzed, with no protective gel layer formed, resulting in the breakdown of the entire glass network [57]. Calcium and other doped elements, along with phosphorus, are then gradually released into the surrounding media, contributing to the formation of a calcium phosphate layer over the surface of the BG.

For phosphate-based BGs with P_2_O_5_ content > 45 mol.%, the formation of abundant phosphoric acid after hydrolytic degradation often leads to increased acidity in aqueous media [58]. This acidity, in turn, accelerates the degradation of phosphate-based BG [59]. As such, the phosphate-based BG undergoes autocatalytic degradation. Moreover, these glasses are less likely to induce precipitation of the bioactive HCA layer after degradation, as increased acidity of an aqueous environment favors the precipitation of dicalcium dihydrate, while HCA is prone to dissolve in an acidic environment [60]. In contrast, several invert phosphate glasses (P_2_O_5_ content < 40%) have been reported as bioactive, considering that the degradation media remained neutral or slightly basic to favor the nucleation of hydroxyapatite [61,62,63]. It has also been reported that titanium in an invert phosphate glass plays a vital role in the induction of bioactivity. Tetravalent titanium is found to ionically cross-link the phosphate units, thereby interrupting the glass network and causing a reduction in hydrolyzable P-O-P bonds, leading to reduced solubility of the phosphate glass. Acidification of the surrounding media is therefore impeded, giving rise to the precipitation of bioactive hydroxycarbonate apatite. Moreover, Kasuga et al. reported the formation of a Ti-enriched interfacial layer between 30P-60Ca-10Ti invert phosphate glass and bioactive apatite. The authors stated that the intermediate layer was likely to be a Ti-OH gel layer, and functioned similarly to the Si-OH gel layer on silica-based bioactive glass to facilitate apatite nucleation [64].

#### 2.1.3. Borate-Based BG

It was at the beginning of the 2000s that borate-based BG caught the attention of researchers in the field of bone tissue engineering [65]. The glass network of a borate-based glass contains trigonal, planar BO_3,_ and tetrahedral BO_4_ at the same time. As shown in Figure 1C, vitreous B_2_O_3_ only contains BØ_3_ units, where all three oxygens are bridging oxygens in between two boron atoms, i.e., B-Ø-B. With the addition of metal oxides, trigonal planar BØ_3_ converts into a tetrahedral BØ_4_^−^ unit composed of four bridging oxygens and one boron bearing negative charge [66]. With all oxygen atoms being bridging oxygens, the network connectivity of the glass network increases. The further addition of metal oxides, however, results in the reversible conversion of BØ_4_^−^ into BØ_2_O^−^, where non-bridging oxygen emerges. The transition of borate units continues with an increasing number of metal oxides added into the network, leading to the formation of BØO^2−^, BØ_2_O^2−^, and eventually, BO^3−^ units. With a lower ratio of bridging oxygens, the glass network becomes depolymerized and the network connectivity is gradually reduced [38]. Along with the changes in the glass network connectivity, a specific physiochemical property of glass (e.g., solubility or strength) usually exhibits a non-monotonous change and maximizes/minimizes as the BØ_4_^−^ content maximizes, corresponding to the maximum network connectivity. This feature is termed “borate anomaly” in the literature [67,68].

As boric acid has rather limited acidity, the degradation of borate-based glass containing oxides of alkali/alkaline earth metal typically creates a neutral or basic pH in the degradation media, which facilitates the formation of hydroxycarbonate apatite over the glass surface [69]. Unlike silicate-based BG, no protective gel layer is formed to protect the inner parts of borate-based glass from further dissolution. Therefore, a borate-based, silicate-free glass converts completely into a bioactive compound enriched with calcium phosphate after immersion in the body fluid [48].

As borate-based BG is fully dissolvable, care must be taken to control the degradation rate of the glass by tuning the chemical formulation. It has been reported that the viability of both murine bone marrow-derived stromal cells and murine pre-osteoblasts-like cells decreased with increasing boron concentration in culture media [70,71]. These findings indicate possible hazards due to the excessively rapid dissolution of borate-based BG. While the dissolution rate was determined by the chemical formulation of glass [65], it was also reported that the macroscale architecture of the glass scaffold, which could be accurately defined via AM, may be exploited to modulate the dissolution rate of borate-based BG [72].

### 2.2. BG-Based Polymer Composites

While bioactivity allows the BG to directly bond to bone tissues, the processing of BG into the desired geometry is difficult owing to the low fracture toughness and a high modulus of BG [76]. For this purpose, one strategy is to develop BG/polymer composites, where BG serves as a functional agent that induces bioactivity by releasing elements, and also mechanical reinforcement to improve the stiffness of the resultant material. The rest of the composite is primarily the polymer matrix, which binds the BG together and provides toughness to the construct. The polymer matrix also endows the composite with ductility against brittle fracture, which is an evident drawback of pure BG. In regard to bone tissue engineering, free space must be provided for subsequent bone growth, and thus, requires the polymers to be degraded upon implantation without inducing adverse effects on the biological systems [77].

Depending on the origin, biodegradable polymers are typically classified as natural polymers and synthetic polymers. Natural polymers, which include proteins (e.g., collagen, gelatin, and silk fibroin), polysaccharides (e.g., cellulose, alginate, hyaluronic acid, and chitosan), and polynucleotides, are naturally occurring materials harvested from animals, plants, or microorganisms [78]. Natural polymers show great similarity to the components of the extracellular matrix in human tissues [79]. For instance, type I collagen constitutes 90% of the weight of the organic phase of bones, whereas type II collagen is abundantly found in cartilage [80,81]. Chitosan, though not native to the human body, is structurally similar to glycosaminoglycans, which constitute the extracellular matrix [82]. This compositional similarity makes natural polymers more recognizable by the biological system, thereby lowering the risk of chronic inflammation and cytotoxicity after degradation [83,84].

Nonetheless, the qualities and properties of natural polymers may exhibit batch variations between different sources [79]. Moreover, pathogens and immunogenic/antigenic substances can be transferred from donors to recipients, causing infection or significant foreign body reactions [85,86]. Last, but not least, natural polymers, especially for proteins, are prone to denaturation due to heat, radiation, and chemicals applied during material processing, which causes impaired mechanical and biological performance. Therefore, care must be taken to control the parameters and chemicals used to avoid denaturation of the natural polymer matrix and altered properties of the composites thereof.

Synthetic polymers, in contrast, are artificially produced materials. The most investigated synthetic polymers are aliphatic polyesters, with representative materials including polyglycolic acid (PGA), polylactic acid (PLA), poly-ε-caprolactone (PCL), and their copolymers. These materials degrade mainly via the hydrolytic route. Following water absorption, the ester groups in the polymer backbone are cleaved via hydrolysis to yield oligomers/monomers. These smaller molecules are further metabolized into CO_2_ and water and are eventually eliminated from the body [77]. Compared to naturally derived polymers, synthetic polymers are superior in the consistency of their properties among different batches. To satisfy the clinical requirement for the degradation rate and initial mechanical properties, the ratio among different monomers and the molecular weight of the resultant polymers can be finely configured on-demand [87,88]. Owing to good biocompatibility, excellent processibility, and mechanical properties, synthetic polymers have received approval from the FDA for clinical application [89,90,91].

However, as synthetic polymers do not exist in the biological system, implanted synthetic polymers and their wear debris may invoke a significant foreign body reaction. Most synthetic polymers are hydrophobic, making it difficult for water and protein to be absorbed on the polymer surface, consequently hindering the adhesion of cells [92]. Another safety issue is that CO_2_ is generated after the metabolization of monomers. This is believed to cause increased acidity at the implantation sites, which impairs the survival of both osteoblasts and mesenchymal stem cells, meanwhile stimulating osteoclastic (bone-resorbing) activities [93,94,95,96]. Interestingly, the addition of BG was reported to enhance the surface hydrophilicity, neutralize the post-degradation acidity, and retard the general degradation rate of composites with a synthetic polymer matrix, thereby ameliorating some of the concerns stated earlier [78].

A summary of the structural and mechanical properties of bone, cartilage, BG, and typical BG composites is listed in Table 2. While BG is too brittle to be machined into desired geometries, the presence of a polymer matrix endows the resultant composites with processibility. A wide range of techniques are available to yield composites of desired shapes and, more importantly, an intricate porous structure that determines the mechanical and degradational properties of scaffolds, as well as tissue ingrowth [97]. Gas foaming, freeze-drying, and thermally induced phase separation (TIPS) are frequently applied to induce porosity in the polymer phases. However, control of the shape, interconnectivity, size, and uniformity of pores has been proven difficult using these techniques [98]. For instance, during uncontrolled freeze-drying, the difference in heat transfer rate throughout the solution in a container leads to uneven pore size in the resultant scaffold [99]. Moreover, the use of organic solvents acts as an additional safety concern in TIPS [100]. The use of porogens of well-defined size, shape, and volume allows for better control over the porous structure, yet the on-demand spatial distribution of pores and complete leaching of porogens cannot be guaranteed [100,101]. There is a pressing need for a novel fabrication technique of BG and BG/polymer composites that can precisely configure the geometry and porous structure so as to achieve accurate fitting of the defect site and a well-defined porous structure that satisfies the need for effective tissue ingrowth.

## 3. Additive Manufacturing of BG and BG/Polymer Composites and Their Application to Bone Tissue Engineering

**Figure 2:**
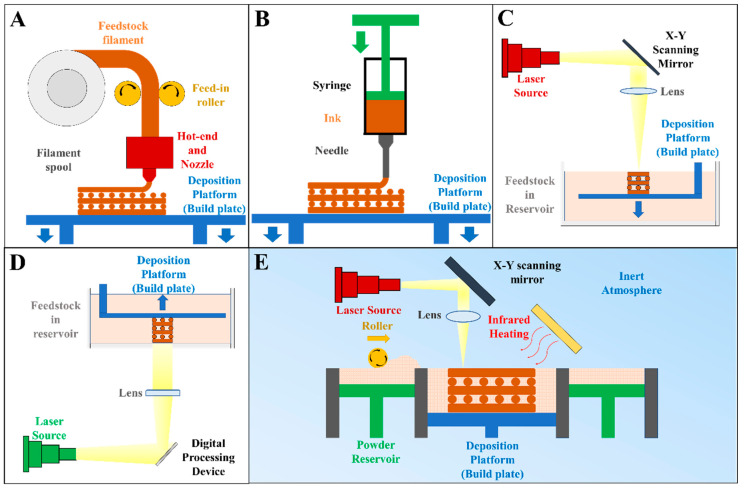
Schematic view of typical AM technologies applied to fabricate pure BG or BG/polymer composites. (**A**) Melt extrusion (also known as fused deposition modeling, FDM); (**B**) direct ink writing (DIW); (**C**) stereolithography (SLA); (**D**) digital light processing (DLP); (**E**) selective laser sintering (SLS, a typical powder bed fusion process).

Additive manufacturing, according to ISO/ASTM 52900:21, refers to a process of “joining materials to make parts from 3D model data, usually layer upon layer, as opposed to subtractive manufacturing and formative manufacturing methodologies” [131]. Depending on how the materials are added to form an integral object, numerous AM technologies have been developed, with melt extrusion, direct ink writing (DIW), vat photopolymerization (including stereolithography and digital light processing), and powder bed fusion being frequently applied to produce BG or BG/polymer composites (Figure 2); these techniques are elaborated upon in the following section.

Following the introduction of AM technologies, this section summarized how additive-manufactured BG or BG/polymer scaffolds have been applied in bone defect healing owing to their ability to fabricate objects with highly complex geometry and intricate porous structures. Published works based on animal studies were reviewed in detail, highlighting the ability of AM to configure the structural properties of resultant objects and to satisfy the need for clinical application, for example, a patient-specific design and the on-demand distribution of multiple functional biomaterials.

### 3.1. AM Technologies Applied to Fabricate BG or BG/Polymer Composite Scaffolds

#### 3.1.1. Melt Extrusion

Melt extrusion refers to a process where molten feedstock is extruded from a nozzle to fabricate an object via on-demand material addition. The feedstock is typically a composite composed of a thermoplastic polymer. It is continuously delivered into a hot end, where heating is applied to melt and liquefy the feedstock. Under the control of a program, the molten feedstock is forced out of the heated nozzle, which travels across the deposition platform to deposit molten feedstock at the specified location. As the temperature drops, the molten extruded materials consolidate, yielding a “sliced” solid layer of the model [132]. Following this, melt extrusion is performed again over the previously deposited layer. With repetitive material deposition in a layer-by-layer manner, a solid object is eventually fabricated.

Most melt extrusion AM devices use feedstock in the form of a continuous filament (Figure 2A). This technology, typically termed “fused deposition modeling (FDM)”, requires that the thermoplastic polymer and BG be homogenized and extruded into filaments beforehand. The incorporation of rigid BG, however, increases the brittleness of filaments, which are prone to breakage when being fed into the hot end [120]. To avoid this, other devices use pellets/powders of a BG/polymer mixture or BG/polymer composites as feedstock, which are melted, and then, propelled either via pneumatic pressure [37] or using a screw [133,134].

Owing to its advantages, including ease of use and the low cost of the device, melt extrusion is one of the most widely applied AM technologies [135], with several bone tissue engineering implants and drug delivery systems fabricated via melt extrusion already cleared for clinical application [136,137]. With the incorporation of BG, the BG/PCL composite scaffolds fabricated via melt extrusion have been reported to have enhanced surface hydrophilicity that improves cell adhesion, as well as the function to enhance in vivo bone regeneration via the elements relseased [138,139].

However, the spatial resolution of melt extrusion-based deposition methods is comparatively low. While nozzles with a small outlet (diameter down to 100 μm) are able to produce fine structures, the force required to extrude molten polymer from a fine nozzle increases dramatically and potentially leads to the buckling/breakage of feedstock filaments. An elevated temperature may reduce the viscosity of polymer melts to ease the extrusion; however, this would be at the expense of more pronounced thermal degradation of the polymer and compromised mechanical properties of the fabricated objects [140].

#### 3.1.2. Direct Ink Writing (DIW)/Robocasting

DIW, also known as robocasting, is another AM technology based on material extrusion. As shown in Figure 2B, the feedstock used in the DIW process is typically a viscous ink, which is extruded through a nozzle and deposited on the deposition platform/previously extruded ink for layer-by-layer material addition. Post-fabrication processing to convert the as-fabricated, semi-solid model into a consolidated object is usually required, with typical post-processing methods including solvent evaporation [121,141], solvent extraction [142], thermal crosslinking [143], photopolymerization [144], and chemical crosslinking [145,146,147].

The key to successful DIW is to obtain inks with ideal processibility. On one hand, the ink shall flow smoothly and uniformly from the nozzle, without pulsating extrusion or clogging of the nozzle. This requires that the BG particles be evenly distributed within the ink without forming large clusters. On the other hand, the extruded material needs to span across the gaps and retain its as-deposited shape without slumping before being converted into a solid. For this purpose, the rheological and viscoelastic properties of ink play a decisive role, as have been reviewed in detail elsewhere [148]. An essential requirement is that inks be shear-thinning such that the viscosity of flowing inks in the nozzle capillary, under a high shear rate, is low to facilitate extrusion. After deposition and the reduction in the shear rate, the viscosity of the ink should be adequately high to maintain its shape. To avoid slumping, M’Barki et al. concluded that the dynamic yield stress of deposited ink should overcome the synergistic effect of gravity and surface tension [149], while Chan et al. considered the product of storage modulus and yield stress as a simple, universal, and effective criteria to predict whether the deposited ink slumps [150]. However, the rheological properties of inks containing BG may not be universally predicted. In most cases, the viscosity of ink typically increases with the addition of BG. For alginate-based inks, the release of Ca^2+^ from BG further induces the ionic crosslinking of alginate, and consequently, increases the viscosity of the ink [151,152,153,154]. Nonetheless, the viscosity of the composite ink may also decline with an increased ratio of BG in the ink, possibly due to the disentangled polymer network of inks and insufficient bonding strength between the BG and the polymer [155,156]. Therefore, the rheological behavior of BG-containing inks needs to be studied to determine the proper BG content within the ink, as well as the parameters of the DIW process.

When compared to melt extrusion, a major advantage of DIW is that a high temperature is not required for liquefying the feedstock. As such, the organic compounds are prevented from undergoing thermal decomposition or denaturation, making DIW a suitable technology to fabricate BG/polymer composite scaffolds composed of natural polymers, drugs, and protein. Zoledronic acid (ZA), an anti-osteoporosis drug with over 20 years of clinical application, was loaded into MBG/PCL inks for the DIW construction of scaffolds [157]. Owing to the low surface area of scaffolds (1.33 m^2^/g), only 28% of the ZA loaded was slowly released into the degradation media after 4 weeks, and it effectively suppressed the osteoclastic differentiation of murine macrophages at the early stage.

Besides polymer composites, DIW has been frequently applied to fabricate pure BG scaffolds based on an indirect process. Using an ink composed of micro- or nanoparticles of BG and a polymer matrix with proper rheological behavior, the DIW process was performed to fabricate green bodies of scaffolds, which are essentially BG/polymer composites. The green bodies were then subjected to heat treatment for thermal decomposition of the organic phase and sintering densification of the residual glass. Specifically, 6P53B [114], 13-93B [158], or 36B-18Si-2P-6Na-8Mg-8K-22Ca [159] were selected as the BGs in studies, owing to their lower crystallization tendency under high temperatures. The obtained BG objects were further spin-coated or dip-coated with MBG or drug-eluting polymers. The coatings endowed the resultant scaffold with increased surface areas for cell adhesion, lower degradation rates [160], as well as the function to locally deliver protein (BMP-2 [161]), a gene (ssDNA [161]), and drugs (dexamethasone [161], HYSA [158]) to stimulate bone regeneration. Wang et al. spin-coated a borosilicate BG with a MoS_2_/PLGA solution. [159]. The coated scaffolds exhibited a photothermal response toward the near-infrared laser, demonstrating great potential for healing bone defects resulting from osteosarcoma-removal surgery.

**Figure 3:**
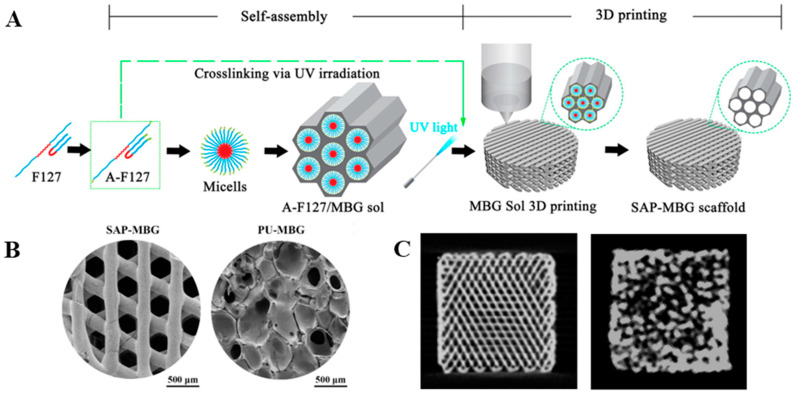
(**A**) Scheme of seamless sol-gel synthesis and additive manufacturing of microporous MBG scaffolds. (**B**) SEM images of seamlessly additive-manufactured scaffold (SAP-MBG) and control group scaffold (PU-MBG). (**C**) Micro-CT images show a cross-sectional view of SAP-MBG (left panel) and PU-MBG (right panel). Reprinted and adapted from reference [119].

A more recent study reported a novel process that seamlessly combined the sol-gel synthesis of MBG and DIW to prepare pure MBG scaffolds (Figure 3) [119]. The key component in this process was acrylated F127, which served to direct the formation of a highly ordered mesoporous structure in sol-gel glass and enabled photopolymerization of the ink upon material deposition. The seamlessly additive-manufactured MBG scaffolds exhibited a highly interconnected macroporous structure with well-defined pore size and pore location while maintaining the mesoporous nature of the MBG. When compared to the control group obtained via the decomposition-sintering densification of MBG over PU foams, the seamlessly fabricated MBG scaffold induced a more pronounced osteogenesis rate of BMSC and more efficient new bone formation in a rat calvaria bone defect. The authors attributed the difference in the morphology of the scaffold to that fact that the concave region at the intersection of extrudates may contribute to the topical enrichment of calcium ions, which positively stimulated osteogenic activity, while the higher interconnectivity in the additive-manufactured scaffold favored tissue ingrowth.

Owing to the mild conditions during fabrication, DIW with ink that encapsulates living cells is technically feasible. Known as extrusion-based bioprinting, this process aims to fabricate living constructs with a well-defined 3D structure to provide a tissue-mimicking structure for cell culture and cell delivery. DIW with bio-inks containing BG has been reported in several works. Owing to its high rigidity and its ability to release doped elements, BG within the bio-ink was reported to modulate the stiffness of bioprint ink, as well as the responses of cells, endowing the bioprinted construct with proper printability. Meanwhile, the BG may induce a specified biological function, depending on its chemical composition. For instance, Zhu et al. used copper-doped MBG to simultaneously enhance the angiogenetic and osteogenesis activities of stromal stem cells within a bioprinted construct [162]. In contrast, Li et al. reported that the silica-based BG nanoparticles within a gelatin–alginate bio-ink inhibited the angiogenic and osteogenic differentiation of BMSC. Instead, the stem cells maintained active proliferation activity as well as stemness, indicating the potential of the bioprinted constructs for stem cell therapy.

**Figure 4:**
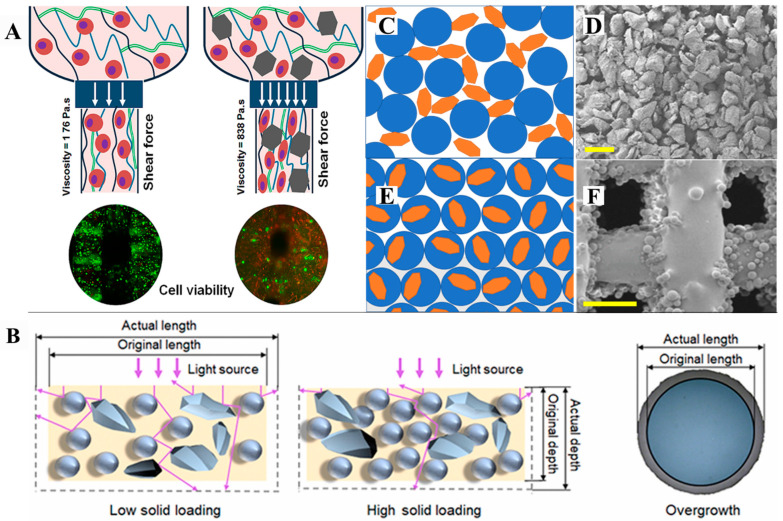
Influence of BG addition on the quality of additive manufacturing. (**A**) BG addition led to increased viscosity and shear force during material extrusion, which impaired cell viability. Reprinted and from references [154]. (**B**) Schematic view of light diffraction due to the presence of BG particles, as well as the consequent variation in cured depth and width of the fabricated products. Reprinted and adapted from references [165]. (**C**–**F**) Scheme and corresponding SEM images of the SLS-fabricated composites using a simple mixture (**C**,**D**) or composite microsphere (**E**,**F**) as feedstock. Orange polygonal particles represent BG, and blue spheres represent polymer. Scale bar = 500 μm. Reprinted and adapted from references [113,129].

While the functions and benefits of BG incorporation in bio-ink were discussed earlier, it is noted that the addition of rigid BG particles may increase the viscosity of the bio-ink, and thereby hinder the spreading of cells (Figure 4A) [163]. In addition, the collision, friction, and steric hindrance of rigid glass particles become more pronounced with increased size and content of the BG, leading to compromised viability and proliferation of cells within the bio-ink [164]. For instance, the addition of 1 wt.% BG microparticles (size = 13–50 μm) into a gelatin–alginate hydrogel ink led to six times greater viscosity at the bioprinting temperature. Consequently, the shear stress during extrusion increased, and the cell viability was markedly lower [154]. However, the addition of BG nanoparticles with an average diameter of 12 nm with doubled concentration (2 wt.%) did not induce evident cell death when compared to the blank gelatin–alginate ink. Nonetheless, with the BG nanoparticle content increased to 5 wt.%, significantly lower cell viability was detected [163].

#### 3.1.3. Vat Photopolymerization

Vat photopolymerization was first developed in the 1980s by Hull as an AM technology [166]. The feedstock used in the vat photopolymerization process is a liquid mixture of photopolymerizable oligomer/monomers, photopolymerization initiator, and other functional additives (e.g., dispersant, dye, and fillers) [166]. Vat photopolymerization begins with the coating of the deposition plane with a thin layer of liquid feedstock. Next, UV or visible light is introduced onto the deposition plate to initiate photopolymerization, converting the fluid feedstock into a layer of polymerized solid pattern. The deposition plate then travels the distance of one layer, allowing the consolidated layer to be coated by the feedstock for the next layer to be photopolymerized. These steps are repeated until the desired model is fully fabricated through layer-by-layer polymerization.

Two types of vat photopolymerization technology have been widely used to fabricate BG or BG/polymer composites. The first one is stereolithography (SLA), during which a light beam(s) moves across the deposition plane and instantly initiates the photopolymerization of feedstock within the light spot (Figure 2C). In the digital light processing (DLP) process, the light is introduced onto the digital micromirror device (DMD), which is an array of micromirrors that can be individually controlled by a program to alternate between the “on” and “off” states. This allows a patterned light reflected from the DMD to irradiate the liquid feedstock, thereby initiating the photopolymerization of a whole layer at once (Figure 2D).

The addition of BG particles is known to affect the rheological and optical properties of feedstock, which may affect the microstructure and properties of the produced parts. For instance, an increased content of BG particles led to higher viscosity of the feedstock [167,168]. As such, a longer time was required for the liquid feedstock to cover and level on the deposition plate, otherwise contributing to the uneven thickness of the polymerized layer. Meanwhile, the presence of BG also affects the photopolymerization behavior of feedstock. A higher concentration of BG may intensify the scattering of light within the feedstock, and thus, alter the depth and width of the polymerized structure, leading to a compromised precision of the manufacturing process [165,169] (Figure 4B). Last, but not least, the influence of BG on photopolymerization kinetics shall not be ignored. Par et al. reported a decreased degree of polymerization with increased BG content in photopolymerizable resin, and attributed this effect to the electron transfer to the oxides in BG [170,171]. A lower degree of polymerization and more residual oligomers could lead to lowered mechanical properties of the produced objects and cytotoxic monomer residuals, thereby jeopardizing the safety of the obtained composites for biomedical application.

Two cases reported the use of DLP to prepare BG scaffolds in an indirect manner (green body fabrication–sintering). Su et al. fabricated gyroid scaffolds composed of 45S5 Bioglass^®^ and biphasic calcium phosphate (BCP) [167]. During sintering, the BG reacted with BCP and hindered the densification of scaffolds, resulting in greater microporosity of the scaffolds. Meanwhile, the reaction products, which include CaSiO_3_ and various sodium calcium phosphates, were more reactive than pure BCP in inducing in vitro hydroxyapatite precipitation. Xu et al. used photopolymerizable slurry-like feedstock containing AP40mod glass-ceramic as a feedstock to fabricate BG-ceramic scaffolds via DLP [172]. With endothelial progenitor cells (EPC) and BMSC seeded onto the scaffolds at an optimized ratio (EPC:BMSC = 2:1), more efficient formation of blood vessels and bone was observed at the implant site, suggesting the potential of the DLP-fabricated AP40mod scaffold as a platform for cell-aided bone defect repair.

#### 3.1.4. Powder Bed Fusion

As the name suggests, powder bed fusion is an AM process where feedstock in the form of loose powders is thermally fused into an integral part. As shown in Figure 2E, the process begins with the spreading and rolling of the feedstock powders over a horizontal building platform to obtain a thin powder layer of uniform thickness. A beam of laser or electron is then introduced onto the powder layer. As the beam scans over the powder layer, the irradiated and heated powders fuse to form a sliced cross-section of the model. The building platform then descends by the thickness of the powder layer, and the abovementioned powder spreading–laser scanning is repeated until the desired object is completely fabricated. The entire process is typically performed in a closed chamber with an inert atmosphere to avoid oxygen-aided thermal decomposition of the materials [173].

Among the powder bed fusion technologies developed, selective laser sintering (SLS) has been widely recruited to produce BG and its composites. This technology utilizes a CO_2_ laser beam as a heat source to partly melt the feedstock powders for fusion. Regarding the fabrication of BG/polymer composites, a low-power (<5 w) laser of high scanning speed (100–1000 mm/s) is typically used, and the SLS process occurs in an inert atmosphere. This configuration is considered to prevent thermal degradation and oxidation of the polymer phase. The feedstock powders are most commonly prepared by blending the fine particles of the BG and the matrix polymer [174]. This process typically yields a highly rough and porous surface over the fabricated composites (Figure 4C,D). In comparison, when composite microspheres or polymer-coated BG particles serve as feedstock, the BG and polymer are more closely integrated at the interface, and the surface of the obtained composites is much smoother (Figure 4E,F), contributing to reduced stress concentration and enhanced mechanical properties of the SLS-fabricated objects [175].

As the sintering of the polymer phase has a low demand for energy input, the transient heating generated by the low-powered, fast-scanning laser may offer energy that is sufficient for polymer sintering without thermally decomposing other organic compounds. Using composite microspheres containing 10 wt.% MBG nanoparticles, dexamethasone, and PLLA, Sun et al. prepared dexamethasone-eluting MBG/PLLA scaffold via SLS for bone regeneration [113]. Dexamethasone was continuously released in 4 weeks, leading to evident osteogenesis of BMSC cultured in a dexamethasone-deficient culture medium, and more pronounced formation of blood vessels and bones in animal models. Shuai and colleagues used polydopamine (PDA)-coated MBG as reinforcement in a PLLA-based polymer composite. The coating applied led to strengthened bonding at the MBG/polymer interface, more homogeneous distribution within the polymer matrix, and improved surface hydrophily of the scaffolds, thereby enhancing the compressive mechanical properties of the scaffolds as well as the adhesion profile of MG63 osteosarcoma-like cells [176]. The PDA coating was further exploited as a reductive agent, allowing graphene oxide and Ag^+^ to be (partially) reduced and immobilized over the surface of PDA-MBG [125,174,177]. These works demonstrate the versatility of SLS-fabricated scaffolds containing PDA-MBG for bone regeneration, with enhanced mechanical properties and additional antibacterial functions.

Alternatively, BG particles without polymer could be directly produced into BG-ceramic constructs via SLS. Under laser beam radiation, the heated glass particles develop into a viscous flow to fuse with the surrounding glass particles or the substrate below. As no polymer binder is used, the obtained part does not require a heat treatment (typically pyrolysis) to remove the binder, thereby being free from potential contamination of the residual binder and shrinkage of size. For instance, Cao et al. successfully produced fully amorphous 13–93 BG scaffolds via SLS [178]. To sinter the BG particles (~100 nm), which require a significantly high temperature to be softened, the laser applied had elevated power (5–9 W) and a lower scanning speed (100 mm·min^−1^) compared to the parameters applied in the SLS of polymer composites (e.g., 5 W/40 mm·s^−1^ [129], 0.09–0.2 W/1 mm·^−1^ [113], and 2.3 W/100 mm·s^−1^ [174]). Using the same device, researchers also succeeded in the direct SLS fabrication of 45S5 [179] and 58S BG [180].

Inevitably, the high-powered, slow scanning laser applied during SLS increases the tendency of thermal crystallization of BG, which leads to impaired bioactivity compared to amorphous BG [181,182]. Whether the BG crystallizes after direct SLS depends on the temperature profile, as a high temperature is required to make the BGs sufficiently soft to fuse with each other, and the transient temperature is likely to be higher than the onset temperature of crystallization (T_c_). For instance, it is reported that 45S5 glass scaffolds produced via direct SLS were partially crystallized. This is believed to be related to a narrow sintering window (T_w_, which is approximately 87 °C for 45S5), namely, the difference in temperature between the glass transition temperature and T_c_ [179,183]. In contrast, 13–93 glass has a wide sintering window (~100 °C) and a high T_c_ (~825 °C), and the glass is believed to be softened enough before reaching T_c_ so that glass fusion without crystallization can occur [184,185]. Regardless of the wide T_w_, crystallization/devitrification of an amorphous glass may still occur considering that the transient temperature during laser processing is above the T_c_. Rodrigo-Vázquez et al. reported that crystalline peaks corresponding to pseudowollastonite were detected in the additive-manufactured 62W glass (T_w_ ≈ 150 °C) scaffold [186]. As the 62W glass underwent devitrification after at 1 h of heat treatment under 900 °C, it is believed that the transient temperature during laser processing was much higher than 900 °C to induce the partial crystallization of 62 W glass in a short period of laser irradiation [187]. Therefore, a systematic analysis of the temperature–viscosity profile of BG and meticulous configuration of the SLS processing parameters are required for the direct SLS of a fully amorphous BG.

In summary, numerous AM technologies have been successfully applied to fabricate BG or BG/polymer composite scaffolds for bone tissue engineering. A comparison of these technologies is presented in Table 3.

### 3.2. Application of Additive-Manufactured BG and BG/Polymer Composites in Bone Tissue Engineering

#### 3.2.1. Scaffold with Patient-Specific Design

The significant variance in both the geometry of bones and the demographic characteristics of patients emphasizes the need for patient-specific bone defect treatments that, to a great extent, rely on a customized geometrical design of the material filling the defect. On one hand, anatomical fitting along with a maximized contact area between the defect and the implanted material provides optimal post-implantation stability, which prevents the undesired dislodgement and loosening of the implant. On the other hand, the geometry of implanted material determines the aesthetic aspects of bone defect healing. This point is especially valuable in the repair of craniomaxillofacial bone defects, as the highly unique yet complex geometry of bones conventionally calls for meticulous intraoperative shaping of the implanted material, making the operation more time-consuming and technically challenging. Owing to its outstanding capability to fabricate complex geometries, AM has become a powerful tool for the fabrication of patient-specific implants. Based on medical images, computer-aided design, and finite element analysis, scaffolds may not only end up with a structural geometry that recapitulates the original bone, but also a customized topological design that restores the biomechanical loading behavior of the original bones [23].

**Figure 5:**
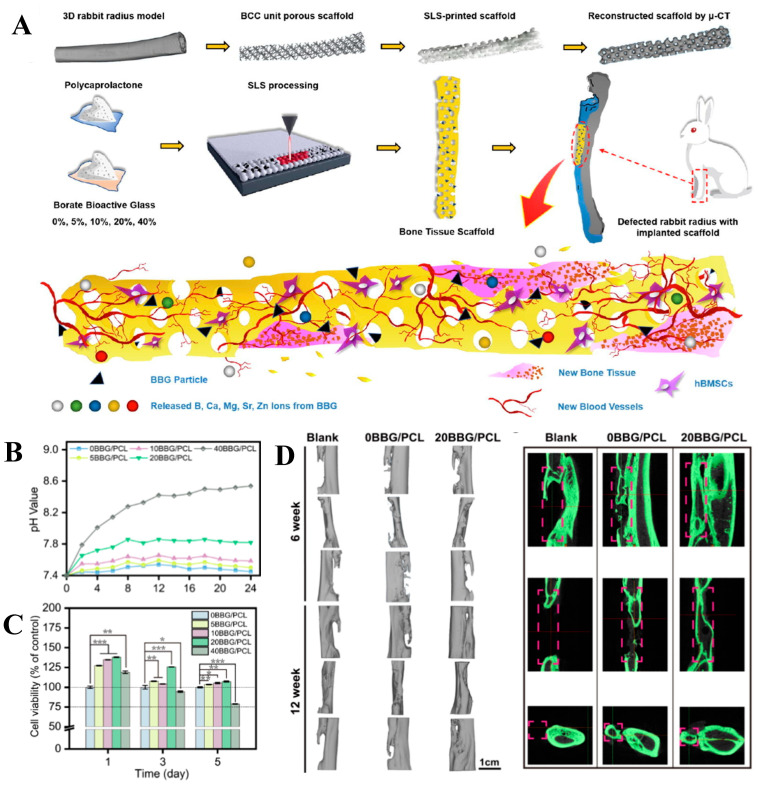
(**A**) Schemes of obtaining a digital model with case-specific outer shape and body-centered cubic units that determines the porous structure, scaffold fabrication, and mechanism of the scaffold to induce bone regeneration. (**B**) Changes in pH of the degradation media after in vitro degradation. (**C**) Cell viability of human bone mesenchymal stem cells co-cultured with different scaffolds. * indicates the statistical significance compared with control group (0BBG/PCL), * *p* < 0.05, ** *p* < 0.01, and *** *p* < 0.001 (**D**) Micro-CT images of rabbit radius after 12 weeks of scaffold implantation. Red boxes indicate the radii of the experiment animals. Abbreviation: BCC, body-centered cubic; BBG, borate-based bioactive glass; PCL, polycaprolactone. Reprinted and adapted from references [188].

Evidence that BG-containing tissue engineering scaffolds with customized geometry led to efficient bone defect healing in animal models was recently reported by Han et al., who prepared a borate-based BG/PCL composite scaffold with a case-specific design via SLS (Figure 5) [188]. To recapitulate the geometry of actual bones, the radii of rabbits were subjected to CT scanning, and the resultant images were converted into 3D models. A Boolean crossover operation between the 3D radii models and porous body-centered cubic units was then performed, producing 3D models with customized geometries and a microporous structure capable of inducing bone ingrowth. Using a mixture of borate-based BG microparticles and PCL powders as feedstock, customized tissue engineering scaffolds were fabricated via SLS and finally implanted into bone defects in rabbit radii. When compared to the control group, where the osteotomy sites were left blank, the presence of customized scaffolds, regardless of the material composition, induced bone regeneration into the interconnecting pores while following the geometry of the customized scaffolds. Moreover, scaffolds containing 20 wt.% BG were most effective at inducing osteogenesis and angiogenesis at the defect site, while the growth of fibrotic tissues (a sign of a foreign body reaction) was minimal around the scaffold, possibly owing to the optimal dosage of ions released by the scaffolds. The authors also emphasized the role of BG content within the composites, reporting that a BG content of 40 wt.% resulted in reduced viability and ALP activities in human BMSC, which may be attributed to the excessively high pH of the extracellular environment after the degradation of scaffolds.

#### 3.2.2. Scaffold with the On-Demand Spatial Distribution of Biomaterials

The flexibility of AM can also be utilized to control the spatial distribution of materials within a single object, yielding bone tissue engineering scaffolds with heterogeneous porosity, and thus, tailored mechanical properties and degradational behavior in different regions. More recently, the development of multi-material and multi-disciplinary/hybrid AM has brought further possibilities to combine different biomaterials into a single object, unchaining the potential of a tissue engineering scaffold that is conventionally limited to a single material composition. Here, we introduced two cases where multi-material AM of BG-containing materials was performed to fabricate scaffolds with the heterogeneous spatial distribution of different biomaterials.

**Figure 6:**
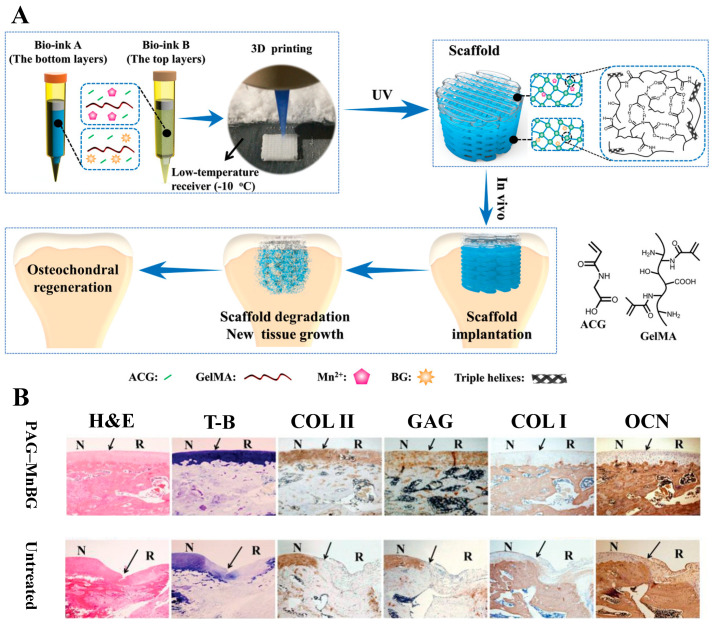
(**A**) Scheme of fabrication of a dual-module hydrogel scaffold intended for osteochondral regeneration. (**B**) Histological staining by hematoxylin and eosin (H&E), toluidine blue (T-B), as well as immunohistochemical staining of chondrogenesis-related antigens (COL II, type II collagen; GAG, glycosaminoglycan) and osteogenesis-related antigens (COL I, type I collagen; OCN, osteocalcin) in sections of rat knee, 12 weeks after implantation. The black arrows point to the margins of the normal and undamaged cartilage (N) and cartilage repaired after scaffold implantation (R). Reprinted and adapted from reference [117].

Degenerative disease of the joints results in damage to the articular cartilage and, if not treated promptly, defects in the subchondral bone. The distinctive characteristics of the two neighboring tissues call for a dual- or multi-component tissue engineering construct that facilitates the regeneration of cartilage, subchondral bone, and, ideally, the interfacial tissue (calcified cartilage) in between [189]. For this purpose, Gao et al. reported the use of DIW to fabricate dual-module scaffolds (Figure 6) [117]. The polymer phase was composed of poly(N-acryloyl 2-glycine) (PACG) and methacrylated gelatin (GelMA). By tuning the concentration of the two polymers, inks with tailored mechanical properties and degradation rates were obtained to satisfy the need for cartilage regeneration (soft matrix, rapid degradation) and bone regeneration (stiff matrix, slow degradation). Furthermore, MnCl_2_ and MBG nanoparticles were selectively loaded into the optimized inks for cartilage repair and bone regeneration, respectively. Single-material scaffolds prepared using Mn^2+^-releasing inks effectively upregulated the chondrogenesis-related genes of hBMSC, whereas those prepared with BG-containing inks induced more pronounced osteogenesis. Next, the two functional inks were used to fabricate the dual-module scaffolds via DIW, with the chondrogenic module deposited on top of an osteogenic module. Following implantation into rat knees, histological sections showed that both the cartilage and subchondral bone regenerated robustly. This work not only demonstrates the potential of the dual-layered scaffold in the repair of cartilage/bone defects at the articular joints, but more importantly, emphasizes the utility of AM to fabricate multi-material, multi-structural constructs intended for multi-tissue regeneration.

**Figure 7:**
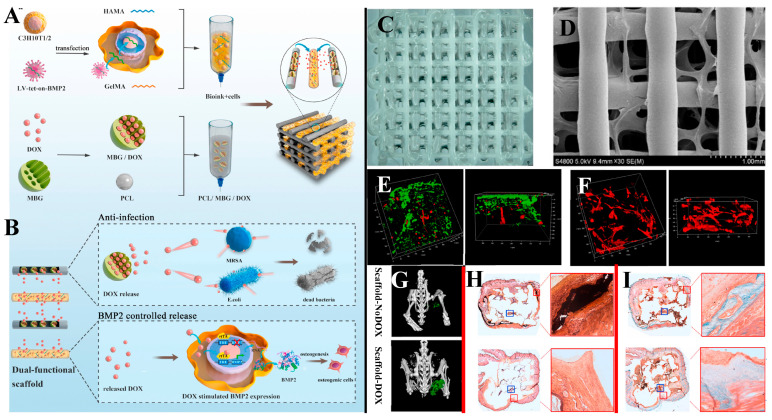
(**A**) Scheme of the preparation of inks and fabrication of hybrid construct. (**B**) Scheme of doxycycline release to suppress bacterial infection and control BMP release. (**C**,**D**) Photograph and SEM image of the hybrid construct. (**E**,**F**) Confocal immunofluorescence images displaying the distribution of murine mesenchymal stem cells (red) and methicillin-resistant *Staphylococcus aureus* (green) in scaffolds with no doxycycline loading (**E**) and scaffolds containing doxycycline (**F**). (**G**) Reconstructed micro-CT images displaying ectopic bone formation (highlighted in green) in nude mice 6 weeks after scaffold implantation. (**H**,**I**) Histological staining of the implantation site to indicate new bone formation, based on Von Kossa staining (**H**) and Safranin O/Fast Green staining (**I**). Red boxes indicate tissues adjacent to remaining scaffolds that are examined under higher magnification. Reprinted and adapted from reference [190].

The incorporation of BG into bioprinting ink enables the responses of living cells (e.g., the maintenance of stemness [163], the upregulation of proliferation, and osteogenic differentiation [162]) within a 3D construct to be modulated. Nonetheless, the increased stiffness of the matrix, collision/friction between cells and rigid BG particles, and the increased shear stress within the flowing ink, which are caused by the presence of a rigid BG within the bio-ink, have been shown to impair cell viability [154]. This conflict was smartly resolved in a recent study [190]. As shown in Figure 7, the authors employed MBG/PCL as the feedstock for the FDM fabrication of a porous 3D framework. Once a layer of the framework was fabricated, bioprinting with a BG-free bio-ink was seamlessly performed. Thus, the MBG modulated the cellular responses “remotely” through its degradation products, while the cells were protected from the adverse effect due to the presence of rigid BG particles within the bio-ink. As a proof-of-concept, the authors designed 3D scaffolds where the degradation of MBG triggered functional expression of the encapsulated cells. The stem cells within the bio-ink were transfected with a lentiviral vector harboring Tet-on-BMP2, thereby enabling controlled transcription of the downstream BMP2 gene in the presence of doxycycline, which was loaded into MBG embedded in the MBG/PCL framework. The results showed that doxycycline, a wide-spectrum antibacterial agent, was continuously released from the MBG/PCL framework and inhibited the growth of pathogens typically related to orthopedic surgeries, thereby rescuing murine stem cells from suppressed survival and proliferation in the presence of MRSA. Meanwhile, BMP-2 transcription was dramatically enhanced in stem cells cultured with doxycycline-eluting MBG/PCL, thereby enhancing the osteogenic activities of stem cells. The hybrid constructs were found to induce ectopic bone formation after subcutaneous implantation, with a greater amount of bone and fewer bacteria present in the surrounding tissues relative to the control group (MBG without doxycycline uptake). With the ability to simultaneously stimulate bone regeneration and prevent bacterial infection, the hybrid construct offers a promising solution to the clinical treatment of large-sized bone defects where bacterial infection typically impairs the efficacy of therapy.

## 4. Perspectives on Future Research

The case studies elaborated upon in Section 4 demonstrate the value of AM in scaffold fabrication, which includes the ability to prepare BG or BG/polymer composite scaffolds of the desired shape, the on-demand spatial distribution of biomaterials, and a well-defined porous structure. These structural features play important roles in determining the physiochemical properties of scaffolds that affect their performance in clinical bone defect healing. The relationships between structure, physiochemical properties, and biological function will be, in our view, a key focus of future research on the AM of BG and its composites. This requires the development of AM technologies that are compatible with BG or BG/polymer feedstocks. These advancements are anticipated to remove the technical barrier of fabricating tissue engineering scaffolds with complex structures, which essentially determines the biological function of the scaffolds to realize safer, more effective, and more patient-specific therapy. Here, we summarize our perspectives on how future studies may be directed in these areas.

### 4.1. Toward a Higher Spatial Resolution

A higher spatial resolution of the manufacturing process enables precise tuning of the structural properties of a BG-containing tissue engineering scaffold. Spatial resolution down to the submicron and even the nanometer scale is useful in the precise control of porous structures, which enables the degradation profile of the BG to be finely adjusted. Moreover, the high-precision manufacturing process imparts cell-sensible topological cues to the surface of scaffolds; these topological cues can be exploited for the rapid (within several hours of contact) induction of cell morphology, and afterward, cellular responses, presumably before a critical concentration of ions are released through BG degradation [191].

**Figure 8:**
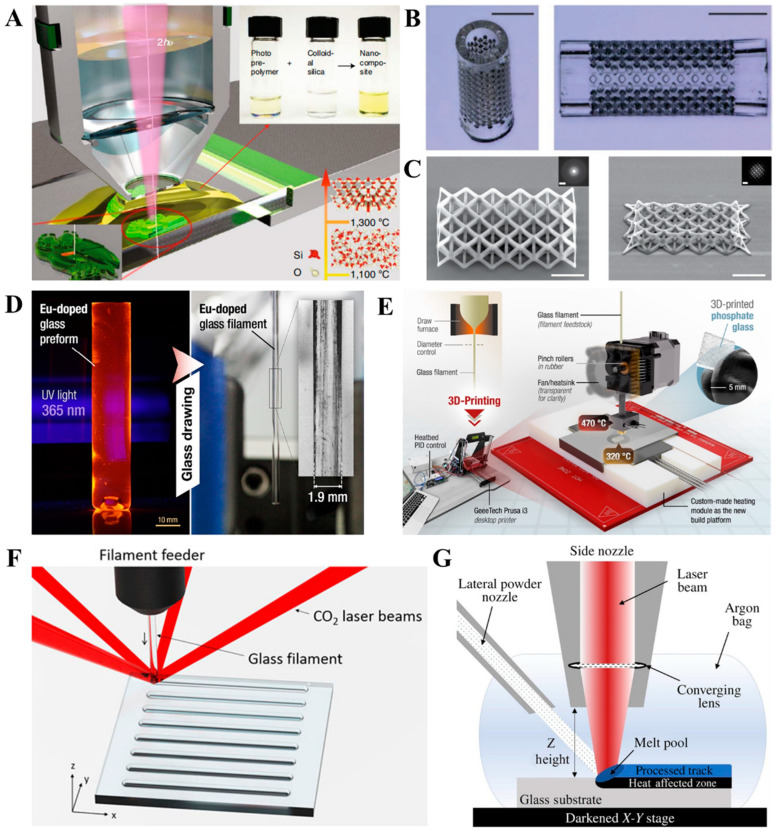
(**A**) Scheme of two-photopolymerization to produce (a green body of) silicate-based glass [193]. (**B**) Photographs of silicate-based glass object produced via 2PP. Scale bar = 500 μm [192]. (**C**) Scanning electron microscopy image of silicate-based glass objects produced via 2PP. Scale bar = 20 nm (image) and 1 nm (inset) [193]. (**D**,**E**) Photographs of phosphate glass preform, and a scheme of direct FDM of phosphate glass [196]. (**F**,**G**) Scheme of laser-assisted direct deposition of molten glass using (**F**) glass rod [197] and (**G**) jets of glass particles [199] as feedstock. Reproduced from references listed above.

Recently, the two-photon polymerization (2PP) technique has been successfully applied in the fabrication of glass with fine structures at the submicron scale, catching the interest of researchers. During the 2PP process, the photopolymerization initiator is attacked by two photons, generating free radicals in a highly localized region near the laser focal spot to initiate the polymerization process, thereby achieving a high spatial resolution of the manufacturing process (Figure 8A). Based on a 2PP AM-thermal debinding and sintering process, Kotz et al. prepared micro-structured models, such as a micro-lens and filtering elements with approximately 55-μm pores (Figure 8B) [192]. Further optimization of the feedstock pushed the resolution of the 2PP process to the sub-200 nm scale (Figure 8C) [193]. With the silica nanoparticles replaced by BG nanoparticles, it is possible to fabricate bioactive BG or BG/polymer scaffolds with submicron structures, which potentially enables more complex and precise control of the physiochemical properties and biological functions of the resultant scaffolds.

### 4.2. Binder-Free AM of Pure BG Objects

Owing to their direct bone-bonding ability, pure BG scaffolds continue to hold great research interest for bone defect treatment. Regarding the AM of pure BG parts, the “indirect AM”, during which the additive-manufactured BG/polymer green bodies are subjected to binder removal and glass sintering at high temperatures, remains the most commonly applied routine. Nonetheless, this process is not only tedious, but is also linked to numerous issues. During sintering, the as-fabricated composite scaffolds undergo significant shrinkage, leading to distortion of the porous structure and deviation in its size [147]. Moreover, the high temperature applied results in the generation of cracks [194] and carbonaceous residuals that are difficult to thermally decompose [195]. In the context of bone tissue engineering, these problems may jeopardize the geometrical fidelity, load-bearing capacity, and biocompatibility of the resultant BG scaffolds. As a result, the effectiveness of bone defect healing is significantly compromised.

Recently, the direct deposition of molten glass has been reported in several studies. Zaki et al. investigated the melt extrusion of phosphate glass with low glass transition temperatures (Figure 8D,E). The melt-quenched phosphate glasses were thermally drawn into a rod-like preform with a diameter of 1.90 mm. A desktop FDM 3D printer equipped with a high-temperature (~500 °C) extruder and build plate was used to additive manufacture pure phosphate glass models in FDM mode, with the layer resolution reduced to 100 μm [196]. In another work, Liu et al. investigated the melt deposition of silica glass, a material typically requiring high temperatures (>1000 °C) to process [197,198]. As shown in Figure 8F, the feedstock was a fused silica glass filament with a diameter of ~196 μm, while four CO_2_ laser beams served as an energy source, focusing on the tip of the filament to locally melt the glass. With fine-tuning of the laser power and the speed of feeder movement, the width of the deposited line was controlled at 240–330 μm, and a 10.5 × 3.5 × 21-mm^3^ (L × D × H) prism with no built-in stress was successfully produced. While the filaments with a diameter of 200–1900 μm required a long time to melt and led to a slow material deposition rate (100 mm/min) applied in the process, Spirrett et al. developed a system that continuously jets fine glass powders (D_50_ = 45 μm) onto a building platform, followed by irradiation with a continuous-wave, ytterbium-doped fiber laser (Figure 8G) [199]. After optimization of the laser power and the glass feeding rate, the laser scan speed was increased to ~700 mm/min, which significantly improved productivity. When compared to earlier works on the melt deposition of glass, the technologies developed in these recent studies seem superior in terms of spatial resolution [198]. Although none of these studies used BG as a raw material, it is possible that by meticulously tailoring the glass formula, BGs with desired biological functions, along with proper thermal and rheological properties, could be acquired to gain compatibility with these novel methods.

### 4.3. Scaffold for the Regeneration of Multiple Tissues at the Bone Defect

The healing of bone defects, especially those caused by trauma, is far more complex than a simple regeneration of bone. Typically, a bone defect involves damage to the bones as well as the surrounding bone-attaching tissues, including the periosteum [200], articular cartilage [201], and ligaments [202]. Therefore, complete healing of the bone defect requires the regeneration of multiple tissues affected and, more importantly, the restoration of a biological bonding of different tissues at their interfaces.

For this purpose, multiple “modules” are expected to be integrated into a single scaffold, with each module mimicking the material composition, microstructure, and cell phenotypes, which are selectively determined to upregulate the regeneration of the targeted tissues. A more challenging issue is constructing interfaces between different modules. It is at the limited region close to the interface that the abovementioned properties display a gradient transition, which prevents an abrupt change in the mechanical properties, thereby ensuring an effective load transfer and mechanical stability without stress concentration [202]. Preferably, different modules should be fused or chemically linked to each other at the interface, which offers stronger bonding strength compared to a stratified structure where different modules are simply stacked together [203].

AM with BG or BG/polymer composites offers an opportunity to satisfy the abovementioned requirement. Because of its high flexibility, AM has long been exploited to produce functionally graded materials. With the spatial distribution of various feedstocks, a monolithic scaffold with gradient porosity and material composition is yielded, with the degradation rate, permeability, and load-bearing capacity locally tuned [204,205,206]. Thus, the possibility further multiplies with BG added into the feedstock. As the type and ratio of functional elements in BG can be flexibly tuned, BGs with variable degradation rates and element release profiles can be obtained through this approach, which enables selective stimulation of the regeneration of different targeted tissues. Finally, the advent of multi-material AM, as well as hybrid AM, further pushes the boundaries of complexity in the composition and structure of BG-containing scaffolds [117,190]. With recent studies reporting the function of BG in stimulating the regeneration of tendons [207] and cartilage [208], additive-manufactured scaffolds doped with different BGs seem to hold much potential for the regeneration of multiple tissues in bone defects.

## 5. Conclusions

The current study offers an overview of additive-manufactured BG or BG/polymer composites as bone tissue engineering scaffolds. AM technologies based on melt extrusion, DIW, vat photopolymerization, and powder bed fusion have been successfully applied to process feedstock containing BG and polymeric binders. With the selection of AM for fabrication, the resultant BG or BG/polymer composites present well-defined geometries and intricate porous structures. The BGs within the additive-manufactured parts maintained their biological function (e.g., upregulating osteogenesis inducing bone-binding bioactivity), while the well-defined shape, size, and porous structure satisfy the need for anatomic fitting of the bone defect and effective bone tissue ingrowth. Specifically, the advent of bioprinting technology allows, for the first time, a viable construct containing BG to be prepared, showing significant potential for stem cell-based therapy for bone defects.

A unique advantage of AM is that it allows a scaffold with a complex shape, intricate porous structure, and even multiple material compositions to be fabricated with high-precision. In this regard, we have proposed three directions that call for future research: improving the resolution of the AM process to a submicron scale, applying binder-free AM technologies to fabricate pure BG objects, and fabricating multi-module scaffolds that stimulate the regeneration of multiple tissues in the bone defects. With more effort applied to these research areas, additive-manufactured BG-containing scaffolds will undoubtedly become more valuable to induce the safe and efficient healing of bone defects.

## Figures and Tables

**Table 2:** Summary of mechanical properties, structural properties of bone, cartilage, and typical BG and BG/polymer composites fabricated via different methods.

Materials	Category	BG Weight Fraction	Structural Properties (D = Dense, P = Porous)	Fabrication Methods	Maximum Stress (MPa)	Elastic Modulus (MPa)	Strain at Maximum Stress (%)	Significance as Biomaterials for Bone Tissue Engineering	Refs.
Cortical Bones	Native Tissue	-	-	-	120–240 (C, human femurs and tibias)	10,000–22,000 (C, human femurs and tibias)	-	-	[102]
Trabecular Bones	Native Tissue	-	-	-	13.57 ± 3.1 (C, human femoral head) 1.6–4.5 (C, human mandibular condyle)	876.8 ± 331.6 (C, human femoral head) 127–431 (C, human mandibular condyle)	-	-	[103,104]
Cartilage (human patellofemoral groove)	Native Tissue	-	-	-	-	0.581 ± 0.168 (normal to the articular surface) 0.854 ± 0.348 (parallel to the articular surface)	-	-	[105]
45S5	Glass	100	D	Melt casting	500 (C) 42 (T)	60,000 (C)	-	-	[106,107]
Phosphate-based Glass Fiber	Glass	100	D	Melt drawing	1021–1253 (T)	59,000–62,000 (T)	~2	Potential as reinforcement in biodegradable orthopedic devices	[108]
Bioglass^®^/PCL	Glass/Polymer Composites	5	P-random Pore Size = 200–400 μm (macropores) Pore Size = ~10 μm (micropores) Porosity = 86.5% ± 0.3%	Porogen leaching	0.12 ± 0.02 (C, yield strength)	1.15 ± 0.32 (C)	-	Porous scaffolds with dual-scale porosity were obtained via porogen leaching–solvent extraction Bioglass^®^ outperformed hydroxyapatite in maintaining superior cell viability of murine osteoblast-like cells	[109]
BPSG (Si80-P5-Ca15)/PLLA	Glass/Polymer Composites	30	P-random Pore Size: ~400 μm Porosity = 71% ± 2%	Porogen leaching	4.2 ± 2 (C)	81 ± 4 (C)	-	Increased content of MBG compensated for the increased acidity due to PLLA degradation while inducing more pronounced new bone formation in animal studies (bone defect at rabbit femoral head)	[110]
Bioglass^®^/PDLLA	Glass/Polymer Composites	30	P-random Porosity = 93–94%	TIPS	0.06 ± 0.03 (C)	2 ± 1 (C)	-	In vitro degradation behavior of BG/PDLLA scaffolds within 600 days was elucidated	[111]
Phosphate-based BG (P50-Ca40-Ti10)/PLLA	Glass/Polymer Composites	30	P-random Pore Size: 190 ± 120 μm Porosity = 78.8% ± 0.35%	Solid-state gas foaming	~1.2 (C)	6.19 ± 0.45 (F)	-	Increased content of phosphate-based BG contributed to a higher weight fraction of rigid BG particles and reduced pore size within composites, both contributing to enhanced mechanical properties of composite scaffolds	[112]
MBG (Si70-Ca30)/PLLA	Glass/Polymer Composites	10	P-0/90 grid-like Width of solid raster = ~500 μm Length of pore side =450–500 μm	AM-SLS	1.5 (C)	25 (C)	~18 (C)	Enhanced proliferation of mBMSC Supported calcium deposition of mBMSC in a dexamethasone-deficient osteogenesis induction medium Promoted both osteogenesis and angiogenesis in a rat calvarial bone defect	[113]
6P53B (Si-based BG)	Glass	100	P-0/90 grid-like Width of solid raster = ~100 μm Length of pore side = 450–500 μm Porosity = 60%	AM-DIW	136 (C, parallel to pore channels) 55 (C, vertical to pore channels)	~2000 (C)	-	Heterogeneous pore sizes in different regions of the scaffold Compressive strength remained superior to that of cancellous bone after 3 weeks of in vitro degradation	[114]
13–93 (Si-based BG)	Glass	100	P-0/90 grid-like Width of solid raster = 330 ± 10 μm Length of pore side = 300 ± 10 μm Pore height = 150 ± 10 μm Porosity = 47% ± 1%	AM-DIW	86 ± 9 (C)	13,000 ± 2000 (C)	~0.8 (C)	Rigid, brittle glass scaffolds became highly deformable after subcutaneous implantation More substantial conversion of BG into HA-SiO_2_ layer after in vivo degradation	[115]
MBG (Si80-P5-Ca15)/PVA	Glass/Polymer Composites	86	P-0/90 grid-like Width of solid raster = ~1000 μm Length of pore side = 1001 ± 48 μm Porosity = 60.4%	AM-DIW	16.1 ± 1.53 (C)	155.13 ± 14.89 (C)	~11 (C)	Substantial HA precipitation on scaffold surface as soon as 1 day after SBF immersion Dexamethasone was loaded into MBG scaffolds, with approximately 70% released in the first 24 h (in vitro degradation)	[116]
BG/PACG-GelMA (BG: Si27-B27-P2-Na6-Mg8-K8-Ca16-Sr6)	Glass/Polymer Composites	1	P-0/90 grid-like Width of solid raster = ~500 μm Length of pore side = ~500 μm	AM-DIW	2.51 (C)	0.249 (C)	~90 (C)	Developed a bicomponent hydrogel system with tunable mechanical properties using PACG and GelMA Hydrogel was functionalized with Mn^2+^ and BG nanoparticles for chondrogenic and osteogenic functions Dual-layered scaffolds (a softer, Mn^2+^-doped upper layer, and a stiffer, BG-doped lower layer) were fabricated via DIW, resulting in the efficient repair of rat knee osteochondral defects	[117]
45S5/PCL	Glass/Polymer Composites	20	P-0/90 grid-like Width of solid raster = 330 ± 10 μm Length of pore side = 400 ± 10 μm Pore height = 321 μm Porosity = 50.87% ± 2.45%	AM-FDM	2.99 ± 0.63 (yield stress)	46 ± 4 (C)	-	High-fidelity fabrication of scaffolds, with porosity and pore size close to the designed value Demonstrated the effect of forced convection/cooling to improve the fabrication fidelity for overhung structures	[118]
MBG (Si85-P5-Ca15)	Glass	100	P-0/60/120 triangular mesh Width of solid raster = ~200 μm Length of pore side = ~180 μm Pore height = ~200 μm Porosity = ~75% P–random Porosity = ~45%	AM-DIW or polymer foam templating	~2.5 (C, AM) ~1.5 (C, foam)	-	~0.75 (C, AM) ~0.65 (C, foam)	Photopolymerizable MBG precursor solution seamlessly used for DIW fabrication of porous scaffolds Greater dissolution rate, higher compressive strength, and greater porosity were achieved in DIW scaffolds compared to those in the scaffolds prepared via polymer foam templating Concave surfaces, a higher Ca concentration, a basic microenvironment, and higher interconnectivity of pores contributed to more pronounced osteogenesis at both in vitro and in vivo levels	[119]
45S5/PLA	Glass/Polymer Composites	1	P-0/90 grid-like Width of solid raster = ~400 μm Length of pore side = ~650 μm Pore height = 200 μm	AM-FDM	12 ± 4 (C)	700 ± 100 (C)	~11 (C)	Compressive mechanical properties of scaffolds matched those of human trabecular bones The incorporation of BG promoted osteogenic differentiation of hADSC	[120]
MBG + Ga (NO_3_)_3_/PCL	Glass/Polymer Composites	30	P-0/90 grid-like Width of solid raster = ~300 μm Length of pore side = 488 ± 53 μm Pore height = ~300 μm	AM-DIW	6.96 ± 1.58 (C)	79.82 ± 16.03 (C)	-	Continuous release of Ga^3+^ inhibited the colonization of methicillin-resistant Staphylococcus aureus (MRSA) and *E. coli*, which suppressed the proliferation and adhesion of MC3T3 murine pre-osteoblasts in a bacterial infection MBG contributed to enhanced osteogenesis of BMSC, while Ga^3+^ inhibited osteoclastic differentiation of bone marrow monocytes MBG + Ga (NO_3_)_3_/PCL scaffolds induced effective bone regeneration in an infected long-bone segmental bone defect (radii of rabbits)	[121]
45S5 (Partially crystallized)	Glass–Ceramic	100	P-0/90 grid-like Length of pore side = ~850 μm Porosity = ~50%	AM-DLP	6.8–22.5 (C)	-	2.5–4.5 (C)	The thermal treatment process and BG solid content were optimized, leading to lower linear shrinkage and improved compressive mechanical properties of glass scaffolds	[122]
13–93	Glass	100	P-0/90 grid-like Length of pore side = ~500 μm Pore height = ~300 μm Porosity = 51% ± 2%	AM-DIW	86 ± 4 (C)	16,000 ± 4000 (F)	~3 (C)	Infiltration of biodegradable polymers into BG of interconnecting porosity led to enhanced toughness, stiffness, and strength (compared to pure BG)	[76]
45S5 (microparticles)/silk fibroin	Glass/Polymer Composites	20 *w*/*v*% in feedstock	P-0/90 grid-like Length of pore side =500–600 μm (macropores) 20–30 μm (micropores) Porosity = ~90%	Cast onto additive-manufactured polymer template	1.21 ± 0.08 (C)	10.35 ± 0.62 (C)	-	BG microparticles outperformed the BG nanoparticles to enhance the compressive modulus of BG/silk fibroin composites BG incorporation favored the proliferation and osteogenic differentiation of hBMSCs	[123]
Silver-doped BG (Si58.6-P7.2-Na1.5-Al4.2-K1.5-Ca24.9-Ag2.1, partially crystallized)	Glass–Ceramic	100	P-0/90 grid-like Length of pore side = 622 ± 139 μm Pore height = ~200 μm Porosity = 70.0% ± 4.9%	AM-FDM followed by thermal debinding	2.84 ± 0.75 (C)	110 ± 60 (C)	~3 (C)	Mineral precipitation occurred over the BG scaffolds, demonstrating in vitro bioactivity of scaffolds The release of silver suppressed the colonization of MRSA	[124]
Silver-doped MBG/PLLA	Glass/Polymer Composites	29	P-0/90 grid-like Length of pore side = ~400 μm	AM-SLS	15.91 (C)	1204.9 (C)	~11 (C)	Ag^+^ inhibited the growth and adhesion of *E. coli* without affecting the survival and proliferation of MG-63 human osteoblast-like cells	[125]
45S5/PCL	Glass–Ceramic	100	P-0/90 grid-like Width of solid raster = ~150 μm Length of pore side = 300 ± 5 μm Pore height = ~120 μm	AM-FDM	9.16 (C, yield stress)	67.4 ± 0.54 (C)	-	The incorporation of BG upregulated the odontogenic gene expression of hDPSCs	[126]
Phosphate-based BG fibers + MgO/PLA (BG: P48-B12-Na1-Mg17-Ca14-Fe8)	Glass/Polymer Composites	18	P–gyroid Pore size = ~500 μm Porosity = 50%	AM-FDM	17.59 ± 3.75 (C)	648.14 ± 81.12 (C)	~7 (C)	Avoided burst pH reduction due to autocatalytic PG degradation Sustained release of calcium and phosphate	[127]
13–93/sodium alginate	Glass/Polymer Composites	33	P-0/90 grid-like Width of solid raster = ~500 μm Length of pore side = 500 ± 24 μm Pore height = ~300 ± 18 μm Porosity = 77.8% ± 2.6%	AM-DIW	16.74 ± 1.78 (C)	79.49 ± 7.38 (C)	~70 (C)	The incorporation of BG with optimized content at 33 wt.% upregulated the proliferation and osteogenic differentiation of rBMSC	[128]
58S/PLDLA	Glass/Polymer Composites	10	P–random Pore Size = ~200 μm Porosity = 26% ± 2%	AM-SLS	2.4 ± 0.6 (F)	79 ± 24 (F)	6.9 ± 3.9 (F)	58S/PLDLA composites displayed cancellous bone-mimetic mechanical properties and good cytocompatibility	[129]
Copper- and magnesium-doped BG (Si54-Ca22-P2-K8-Na6-Mg7-Cu1)	Glass	100	P-0/90 grid-like Width of solid raster = ~200 μm Length of pore side = ~300 μm Porosity = 50.99% ± 1.2%	AM-DIW	109.27 ± 8.18 (C)	-	-	Promoted osteogenic differentiation of mBMSC, and angiogenic functions of HUVECs Suppressed the colonization of *E. coli* and *S. aureus* Promoted bone regeneration in long-bone segmental defects (radii of rabbits)	[130]

Note: C/F/T in maximum stress or elastic modulus denote modes of mechanical testing. C = compression, F = flexure, T = tension.

**Table 3:** Comparison of various AM technologies applied in the fabrication of BG or BG/polymer composite scaffolds.

AM Technology	Feedstock	Advantages	Limitations	Refs.
Melt extrusion (FDM)	A solid powdery mixture or composite filaments of BG particles and thermoplastic polymer	Desktop devices available at low cost Multi-material AM devices are commercially available	A limited resolution of structural feature (>100 μm) High-temperature heating is applied during AM process and filament fabrication Additional cost for filament fabrication equipment Requires supports for the overhung structure	[37,120,138,139]
Direct ink writing (including bioprinting)	Liquid ink homogenized with BG particles	Good compatibility with a wide range of polymers, including natural polymers No high temperature is required during the AM process Available for extrusion-based bioprinting Supports multi-material AM	Meticulous configuration of rheological properties required Post-processing is often needed For bioprinting, the addition of BG impairs cell viability Requires supports for the overhung structure	[117,119,123,162]
Vat photopolymerization (e.g., SLA and DLP)	Liquid photopolymerizable resin homogenized with BG particles	Good resolution (~50 μm) DLP process has a high manufacturing speed	High BG content impairs dimensional accuracy Multi-material AM Requires supports for the overhung structure Potential cytotoxicity of photo-initiator leftover Limited range of available materials	[165,172]
Powder Bed Fusion (e.g., SLS)	Solid powders of mixtures or a composite of BG particles and thermoplastic polymer	Allows direct AM using BG powders as feedstock No supports are required for the overhung structure	High cost of AM device High transient temperature potentially leads to the crystallization of BG and degradation of the polymer	[125,178,188]

## Data Availability

No new data were created in this study. Data sharing is not applicable to this article.

## Abbreviations

AM	Additive manufacturing
BCP	Biphasic calcium phosphate
BG	Bioactive glass
BMP-2	Bone morphogenic protein 2
m/r/hBMSC	Mouse/rat/human bone mesenchymal stem cell
DIW	Direct ink writing
DLP	Digital light processing
DMD	Digital micromirror device
*E. coli*	*Escherichia coli*
EPC	Endothelial progenitor cell
FDM	Fused deposition modeling
GelMA	Gelatin methacryloyl
HA	Hydroxyapatite
hADSC	Human adipose-derived stem cell
HCA	Hydroxycarbonate apatite
hDPSC	Human dental pulp stem cell
HIF-1α	Hypoxia-inducible factor 1 α
HUVEC	Human umbilical vascular endothelial cell
HYSA	Hydroxy-safflower yellow A
MBG	Mesoporous bioactive glass
MRSA	Methicillin-resistant *Staphylococcus aureus*
PACG	Poly (N-acryloyl 2-glycine)
PCL	Poly-ε-caprolactone
PDA	Polydopamine
PDLLA	Poly (DL-lactic acid)
PGA	Polyglycolic acid
PLA	Polylactic acid
PLDLA	Poly (L-co-D, L-lactic acid)
PLGA	Poly (lactic-co-glycolic acid)
PLLA	Poly (L-lactic acid)
PU	Polyurethane
*S. aureus*	*Staphylococcus aureus*
SLA	Stereolithography
SLS	Selective laser sintering
TIPS	Thermally induced phase separation
TNF-α	Tumor necrosis factor α
ZA	Zoledronic acid
